# Characterization and phylogenetic analysis of the complete chloroplast genome of *Amaranthus viridis* (Amaranthaceae)

**DOI:** 10.1080/23802359.2021.1961631

**Published:** 2021-08-09

**Authors:** Dian-Bin Ding, Qi-Hang Pang, Xiao-Jun Han, Shou-Jin Fan

**Affiliations:** a Binzhou Yellow River Irrigation Management Service Center, Binzhou, China; b Key Lab of Plant Stress Research, College of Life Sciences, Shandong Normal University, Ji’nan, China

**Keywords:** *Amaranthus viridis*, plastome, Phylogeny

## Abstract

*Amaranthus viridis* is an important medicinal herb. In this study, the complete chloroplast genome (plastome) of *A. viridis* was repotred. It was a circular molecular of 150,452 bp in length and consists of a large single-copy region (LSC, 83,832 bp), a small single-copy region (SSC, 17,914 bp), and two inverted repeats (IRs, 24,353 bp for each) regions. The overall GC content was 36.6%. This plastome encodes 113 unique genes, including 79 protein-coding genes, 30 tRNAs, and four rRNAs. The phylogenetic tree of 18 Amaranthaceae chloroplast genomes supported that *A. viridis* was closely related to *A. hybridus*.

*Amaranthus viridis* L. is an edible and medical annual herb from Amaranthaceae. *A.viridis* is native to Africa, and now is widespread all over the world (Thomas et al. 2006). In traditional system, *A. viridis* was used to relieve labor pain and as antipyretic in Indian and Nepal (Kirtikar and Basu 1987; Turin 2003). *A. viridis* also has numerous medical uses, such as antihepatotoxic, antiulcer antiallergic, and antiviral actions (Ashok Kumar et al. 2012; Reyad-ul-Ferdous et al. 2015). Moreover, *A. viridis* has a strong capability to accumulate heavy metals (Zou et al. 2006; Ramanlal et al. 2020). When growing on heavy metal contaminated soil, *A. viridis* is a phytostabilizer of Zn and Mo (Ameh et al. 2019). *A. viridis* was also an excellent source of natural antioxidant phytopigments (Sarker and Oba 2019). However, no chloroplast genome resource is available so far for this medicinal herb. In this study the complete chloroplast genome of *A. viridis* was reported for the first time.

Fresh leaves of *A. viridis* were collected from Bingzhou Xiaokaihe Irrigation District (Shandong, China; 37°41′ N, 117°46′ E). A specimen was deposited at Shandong Normal University (shoujin Fan, Email: fansj@sdnu.edu.cn) under the voucher number 20127. DNA was extracted from silica dried leaves using a modified CTAB method (Guo et al. 2020; Wang et al. 2019). Total genomic DNA was used for library preparation and paired-end (PE) sequencing by the Illumina MiSeq at Novogene (Beijing, China). Organelle Genome Assembler (https://github.com/quxiaojian/OGA; Qu, Fan, et al. 2019) was used to assemble the plastome. Annotation was performed with Plastid Genome Annotator (PGA, https://github.com/quxiaojian/PGA; Qu, Moore, et al. 2019), coupled with manual correction using Geneious v9.1.4. In order to determine the phylogenetic placement of *A. viridis*, a Maximum-Likelihood (ML) tree was constructed by RAxML V8.2.10 (Stamatakis 2014) using 1000 rapid bootstrap replicates with the GTRGAMMA substitution model. Two Gomphrena species were selected as outgroups. The alignment of 79 shared protein-coding genes (PCGs) was carried out using MAFFT v7.313 (Katoh and Standley 2013).

The complete chloroplast genome of *A. viridis* (the GenBank accession number: MW679034) was 150,452 bp in length and composed of a large single-copy region (LSC, 83,832 bp), a small single-copy region (SSC, 17,914 bp), and a pair of inverted repeats (IRs, 24,353 bp for each). A total of 113 genes were annotated in this plastome, including 79 protein-coding genes (PCGs), 30 tRNA genes, and four rRNA genes. Among them, nine protein-coding genes (*atp*F, *ndh*A, *ndh*B, *pet*B, *pet*D, *rpl*16, *rpo*C1, *rps*12, and *rps*16) and six tRNA genes (*trn*K-UUU, *trn*G-UCC, *trn*L-UAA, *trn*V-UAC, *trn*I-GAU, and *trn*A-UGC) contained one intron. Two protein-coding genes (*clp*P and *ycf*3) contained two introns. The overall GC content was 36.6%. The ML phylogenetic tree showed that *A. viridis* was closely related to *A. hybridus* (Figure 1).

## Figures and Tables

**Figure 1.**
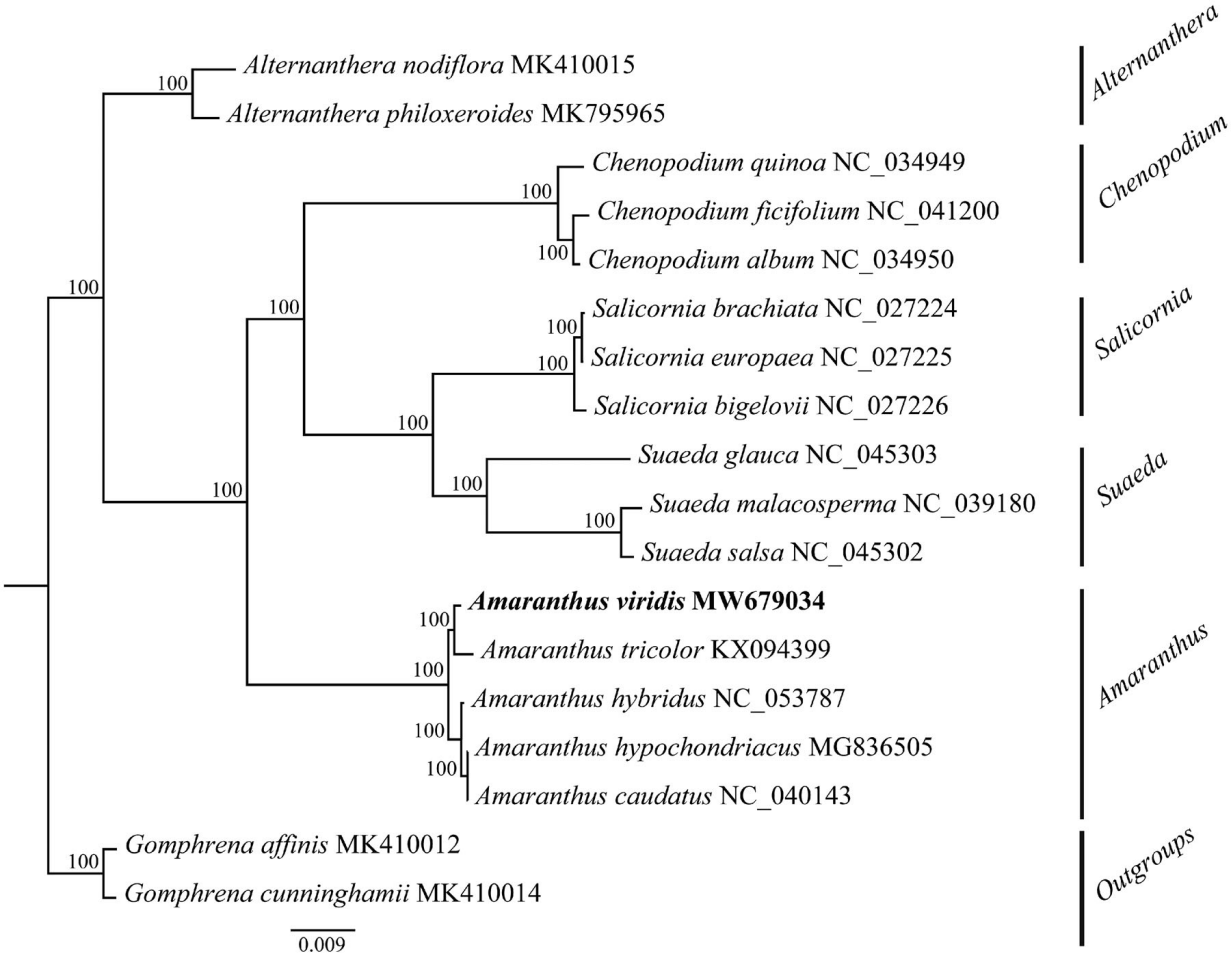
A Maximum-Likelihood (ML) phylogenetic tree based on 18 Amaranthaceae plastomes was shown. Bootstrap support values are shown next to branches.

## Data avaliability statement

The data that support the findings of this study are openly available in GenBank of NCBI at https://www.ncbi.nlm.nih.gov, reference number MW679034. The associated BioProject, SRA, and Bio-Sample numbers are PRJNA720789, SRR14194277, and SAMN18679672, respectively.
